# The preventive effects of *Zataria multiflora* and carvacrol and their co-administration with pioglitazolne on systemic inflammation and oxidative stress induced by paraquat inhalation in rats

**DOI:** 10.22038/AJP.2024.24272

**Published:** 2024

**Authors:** Mahla Mohammadi Mahjoob, Sima Beigoli, Arghavan Memarzia, Javad Ghasemi, Mohammad Hossein Boskabady

**Affiliations:** 1 *Applied Biomedical Research Center, Mashhad University of Medical Sciences, Mashhad, Iran*; 2 *Department of Physiology, School of Medicine, Mashhad University of Medical Sciences, Mashhad, Iran*

**Keywords:** Zataria multiflora, Paraquat, Carvacrol, PPAR-γ agonist, Oxidative stress, Inflammation

## Abstract

**Objective:**

The present study aimed to assess the impact of the aqueous-ethanolic extract of *Zataria multiflora* (ZM), carvacrol (Car), and their co-administration with a PPAR activator, pioglitazone (Pio), on oxidative stress and inflammation induced by paraquat (PQ) inhalation at a systemic level.

**Materials and Methods:**

The rats in the control group were exposed to saline and those of other groups to PQ (54 mg/m^3^) aerosols for 8 times on alternate days. Nine PQ groups were treated with saline, Car (20 and 80 mg/kg/day), ZM (200 and 800 mg/kg/day), Pio (5 mg/kg/day), dexamethasone (Dexa, 0.03 mg/kg/day), and low-dose ZM or Car + Pio for 16 days during the period of PQ exposure (n=6).

**Results:**

Differential and total WBC counts, and malondialdehyde (MDA), interleukin (IL)-10, and tumor necrosis factor (TNF)-α levels were enhanced but catalase (CAT), thiol, and superoxide dismutase (SOD) levels were reduced in the blood in the PQ group (p<0.01 to p<0.001). All measured variables improved in groups treated with both doses of ZM, Car, Pio, ZM + Pio, Car+Pio, and Dexa *vs* the PQ group (p<0.05 to p<0.001). Most variables were more improved in combined treatment groups in comparison with three agents alone. The combination of ZM or Car, and Pio showed an impact on PQ inhalation-induced systemic changes.

**Conclusion:**

The synergistic effect between Pio with ZM or Car indicates that these substances work together to enhance their individual effects.

## Introduction

Paraquat (1,1'-dimethyl-4,4'-bipyridylium) (PQ) is a herbicide that was first introduced in 1882. Paraquat is a very strong and widely used herbicide from the bipyridylium group and is a green liquid (Delirrad et al., 2015). The herbicidal properties of PQ were first described in 1955 and entered the market commercially in 1962, but it is considered lethal to both humans and animals. Poisoning by herbicides used in agriculture is a major health problem in the world (Sittipunt, 2005). The poisonous effects of PQ on the lungs include lung edema, hypoxia, and extensive acute lung injury (ALI) (Gao et al., 2020) and with more exposure, lung damage can become more serious (Chen and Lua, 2000). The pulmonary and systemic toxicity of PQ were examined with inhalation, oral, and intraperitoneal (i.p.) administration models (Amin et al., 2021b; Gohari-Piran et al., 2022). Enhanced intracellular guanosine monophosphate (GMP) and ultimately activated guanylate cyclase were shown with i.p. PQ administration (Giri and Krishna, 1979), and increased fibroblasts in the lung, emphysema, lung fibrosis and pneumothorax were recorded (Kuo et al., 2021). In addition, It has been shown that PQ administration leads to lung and systemic inflammation through increased expression of toll-like receptor 9 (TLR9), interleukin IL-6, (IL)-1β, thromboxane B2 (TXB2) and tumor necrosis factor (TNF) (Huang et al., 2019). The mechanisms of PQ-induced ALI are basically related to the systemic inflammation (Yu et al., 2013). It has been shown that inhalation of PQ leads to an increase in total and differential white blood cell (WBC) and changes in oxidative shown by increasing malondialdehyde (MDA) and reducing thiol levels and superoxide dismutase (SOD) and catalase (CAT) activities (Amin et al., 2021b; Rojo et al., 2007). It was shown that PQ inhalation increases lung fibrosis and other pathological changes, which were associated with raising apoptosis makers like Bax/Bcl2 and caspase-3 expression in an intense ALI model (Rashidipour et al., 2021). 

*Zataria multiflora *or thyme (ZM) is a plant from the Lamiaceae family and is originally from south‐western Asia (Ghorani et al., 2022). ZM has been used in traditional medicine for many purposes, such as treatment of infections, digestion disorders, boosting energy, promoting sweating, increasing urine production, reducing muscle spasms, and relieving pain (Khazdair et al., 2018). Different chemicals including phenolics (carvacrol, thymol and linalool), and non-phenolics (γ-Terpinene, p-cymene and α-Pinene), have been identified in ZM (Ghorani et al., 2022). Carvacrol (Car) is a type of oxygenated monoterpene that is found in large amounts in ZM. Several studies have focused on the anti‐inflammatory potential of ZM and Car (Nakhai et al., 2007). The antioxidant property of this plant is due to the presence of high amounts of thymol and carvacrol (Moosavy et al., 2008). The extract of ZM decreased oxidative stress by trapping free radicals and raising antioxidant factors like CAT, SOD, glutathione (GSH), and reducing factors such as nitric oxide (NO), MDA and lipid peroxidation (Rezaie and Rasouli, 2001). Also, antibacterial (Naghibi et al., 2005), antifungal (Rahimi et al., 2019), and antioxidant (Dashipour et al., 2015) properties of the plant due to the presence of compounds such as Car and thymol were shown (Braga, 2005; Kamatou et al., 2005). The inhibitory effect of Car on cyclooxygenase 2 (COX-2), which inhibits the production of prostaglandin E2, indicates the anti-inflammatory effect of Car (Landa et al., 2009). These compounds affect cell membrane by inducing morphological and permeability changes, leading to the release of the cell contents (Moosavy et al., 2008). 

Peroxisome proliferator-activated receptors (PPARs) are part of the nuclear receptor family. The superfamily of nuclear receptors includes vitamins D, thyroid, retinoic acid and steroids, and peroxisome proliferation activating receptors PPARs. One of the nuclear receptors that interacts with the ligand is comprised of three distinct isoforms, namely α, γ, and δ, each originating from separate genetic sources. PPAR- γ isoform also includes three isoforms γ1, γ2, and γ3, in which mRNA of γ2, and γ3 isoforms are translated into γ1 protein. After connecting the PPAR-γ ligand, it forms a heterodimer with other nuclear receptors called retinoic X receptor (RXR) (Ahmed et al., 2007). PPAR-γ is connected to spaces called peroxisomal replication responsive regions (PPRE) in the promoter of the target gene and regulates the expression of the target gene (Murphy and Holder, 2000).

A combination of glucocorticoid and cyclophosphamide has been recommended to treat PQ poisoning (Zhang et al., 2012). However, there is currently no effective treatment available for managing the complications of PQ poisoning such as lung insults.

The purpose of this study was to determine if ZM extract or Car, either individually or in conjunction with Pio, could protect against PQ-induced systemic damage. Previous studies have shown the treatment effects of both ZM and Car on systemic effects induced by inhaled PQ in rats (Amin et al., 2021a; Amin et al., 2021b). In these studies, animals were exposed to inhale PQ for 8 times during 16 days and were treated thereafter for another 16 days and variables were measured on day 32. However, in this study, rats were exposed to PQ similar to previous studies but treated during the exposure period and variables were examined on day 16. The rationale behind using pioglitazone (Pio) in combination with ZM or Car is based on evaluating their synergic or additive properties. By combining Pio with either ZM or Car, we hypothesized that a synergistic effect may be achieved by Pio with ZM or Car.

## Materials and Methods

### Plant material and extraction

The collection of ZM (Herbarium No. 35314, FUMH) and the preparation of its extract was fully explained in a previous study (Hakimizadeh et al., 2013). 

### Experimental groups

Sixty male Wistar rats (200–250 g) were kept in in Plexiglas cages in the animal house at School of Medicine, Mashhad University of Medical Sciences (MUMS) at standard condition of 22°C temperature, 12 hr light/dark cycle, 54 ± 2% humidity, and water and food *ad libitum* during the experimental time. Experiments were done based on the rules of the ethics committee, MUMS for Animal Experiments (Code: 981733). In addition, animal experiments were performed based on the criteria of the Guide for the Care and Use of Laboratory Animals (NIH US publication 23-68 revised 1985; http://oacuod.nih.gov/regs/guide/guidex.htm). 

The rats were randomized in the following groups (in each group, n = 6):

(1) Rats of the control group were exposed to saline aerosol. 

(2) Rats of nine PQ group, were exposed to PQ (Sigma-Aldrich Chemical Co., St. Louis, MO, USA) aerosol at dose of 54 mg/m^3^ and treated with: (a) Saline (Burleigh-Flayer and Alarie, 1987) as PQ group. (b and c) Two doses of the extract of ZM (200 and 800 mg/kg/day as ZM-L and ZM-H groups, respectively) (Heydari et al., 2021). (d and e) Tow doses of Car (20 and 80 mg/kg/day as Car-L and Car-H respectively). (f) Pio (Samisaz Pharmaceutical Company, Iran) at 5 mg/kg/day as Pio group (Malekinejad et al., 2014). (g) Combination of ZM-L + Pio. (h) Combination of Car-L + Pio. 0.03 mg/kg/day of dexamethasone (Dexa) (Sigma-Aldrich Chemical Co., St. Louis, MO, USA) as Dexa group.

**Figure 1:**
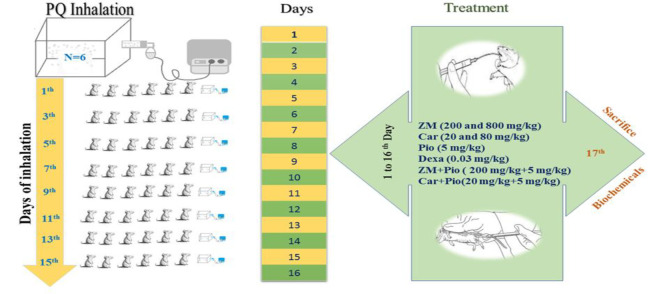
Schematic view of treated groups and animals' exposure to saline (control or PQ (other groups) aerosols. In treated groups, animals were treated with 200 and 800 mg/kg/day *Zataria multiflora* (ZM-L and ZM-H) extract, 20 and 80 mg/kg/day carvacrol (Car-L and Car-H), 5 mg/kg/day pioglitazone (Pio), the combination of ZM + Pio and Car + Pio and dexamethasone (Dexa). Pio was administered by i.p. injection but other agents by gavage for 16 days during PQ exposure period (n = 6 in each group).

 Animals of the control group were exposed to saline and those of the other groups to PQ aerosols, 8 times on alternate days, during 16 days as previously described in detail (Alemán-Laporte et al., 2020). Dexa, Pio, Car and the extract were administered by gavage, but Pio by i.p. injection simultaneous with PQ exposure (Amin et al., 2020) (Figure 1). 

On day 17, animals were sacrificed by i.p. administration of xylazine (5 mg/kg) and ketamine (50 mg/kg) for deep anesthetization (Heydari et al., 2021).

### Measurement of cytokines

Using special enzyme-linked immunosorbent assay (ELISA) kits and according to the recommendation of the manufacturer (Karmania Pars, Kerman, Iran), levels of IL-10 and TNF-α in the serum were evaluated by the method previously described (Cicchese et al., 2018).

### Preparation of blood, and evaluation of differential and total WBC counts and biochemical agents

Blood samples were obtained from the hearts of the animals based on previous reports. Differential and total counts of white blood cells (WBC) were assessed in 1 ml blood (Eftekhar et al., 2018). The other 4 ml of the blood was centrifuged (10 min at 2000 rpm) and serum was collected. Until the estimation of oxidative stress markers and cytokines levels, the serum was kept at -70°C. 

### Measurement of oxidant and anti-oxidant markers

The concentration of oxidant biomarkers including MDA and antioxidants including CAT and SOD activities, and total thiol content in serum were evaluated as explained before (Shakeri et al., 2017).

### Data analysis

Data analysis was performed through one-way analysis of variance (ANOVA) and Tukey's multiple tests. Mean±SEM of the results are provided and statistically significant criterion was p<0.05.

## Results

### Differential and total WBC

**Figure 2:**
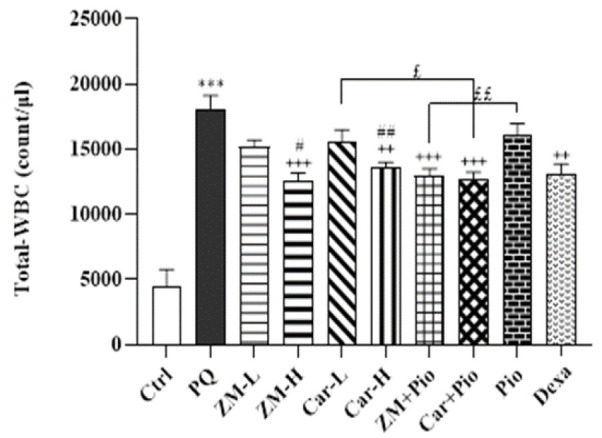
Total number of white blood cells (WBC) in the blood of control (Ctrl) and groups  treated with two doses of *Zataria multiflora* extract (200 and 800 mg/kg/day, ZM-L and ZM-H), two doses of carvacrol (20 and 80 mg/kg/day, Car-L and Car-H), pioglitazone (5 mg/kg/day, Pio) as well as low dose extract + pioglitazone (ZM + Pio) and low dose carvacrol + pioglitazone (Car + Pio), dexamethasone (0.03 mg/kg/day, Dexa).(Number of animals in each group = 6). The results are shown as Mean ± SEM. The results were compared among different groups using ANOVA and Tukey statistical tests. Statistical differences between PQ *vs* control group: *** p<0.001, Statistical differences between PQ *vs* treatments groups: ++ p<0.01 and +++ p<0.001, Statistical differences between combination groups *vs* ZM-L, Car-L and Pio groups: £ p<0.05 and ££ p<0.01, Statistical differences between low *vs* high dose of the extract and carvacrol: # p<0.05 and ## p<0.01.

**Figure 3:**
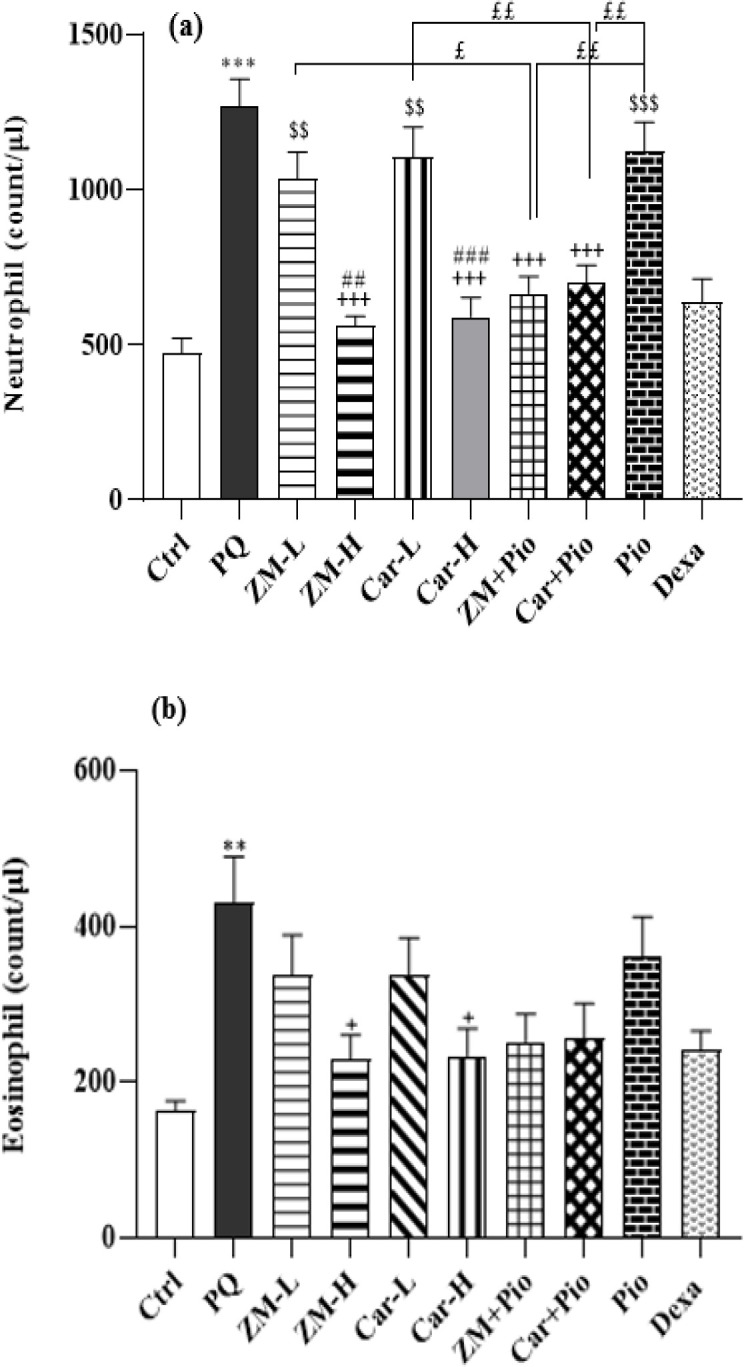
The number of Neutrophil (a) and Eosinophil (b) in the blood of paraquat (PQ, 54 mg/m^3^), control (Ctrl) and groups treated with two doses of *Zataria multiflora* extract (200 and 800 mg/kg/day, ZM-L and ZM-H), two doses of carvacrol (20 and 80 mg/kg/day, Car-L and Car-H), pioglitazone (5 mg/kg/day, Pio) as well as low dose extract + pioglitazone (ZM + Pio) and low dose carvacrol + pioglitazone (Car + Pio), dexamethasone (0.03 mg/kg/day, Dexa). (Number of animals for each group = 6). The results are shown as Mean ± SEM. The results of the groups were compared using ANOVA and Tukey statistical tests. Statistical differences between PQ *vs.* control group; ** p<0.01 and *** p<0.001, Statistical differences between PQ *vs.* treatments groups: + p<0.05, and +++ p<0.001. Statistical differences between Dexa *vs* treatment groups: $$ p<0.01 and $$$p<0.001. Statistical differences between combination groups *vs* ZM-L, Car-L and Pio groups: £ p<0.05 and ££ P<0.01. Statistical differences between low *vs* high-dose groups of extract and carvacrol: ## p<0.01 and ### p<0.001.

**Figure 4:**
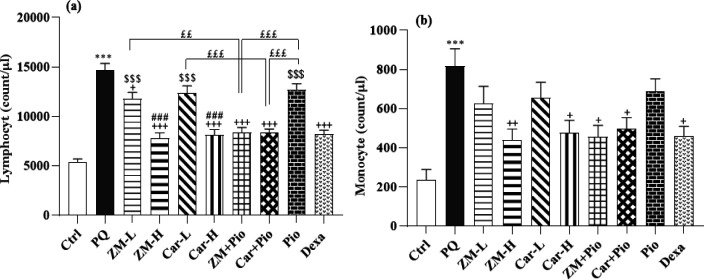
The total number of Lymphocyte (a) and Monocyte (b) in the blood of paraquat (PQ, 54 mg/m^3^), control (Ctrl), control (Ctrl) and groups  treated with two doses of *Zataria multiflora* extract (200 and 800 mg/kg/day, ZM-L and ZM-H), two doses of carvacrol (20 and 80 mg/kg/day, Car-L and Car-H), pioglitazone (5 mg/kg/day, Pio) as well as low dose extract + pioglitazone (ZM + Pio) and low dose carvacrol + pioglitazone (Car + Pio), dexamethasone (0.03 mg/kg/day, Dexa). (Number of animals for each group = 6). The results are shown as Mean ± SEM. The results of the groups were compared using ANOVA and Tukey statistical tests. Statistical differences between PQ *vs* control group: *** p<0.001, Statistical differences between PQ *vs* treatments groups: + p<0.05, ++ p<0.01 and +++ p<0.001, Statistical differences between Dexa *vs* treatment groups: $$$ p<0.001. Statistical differences between combination groups *vs* ZM-L, Car-L and Pio groups: ££ p<0.01 and £££ p<0.001. Statistical differences were between low *vs* high dose groups of extract and carvacrol: ### p<0.001.

All differential (eosinophils, monocytes, and neutrophils) and WBCs counts in the PQ group were higher than the control group (for eosinophil, p<0.01 and for other cases, p>0.001) (Figures 2 and 3). In the PQ group total WBCs, neutrophil, monocyte and lymphocyte counts were significantly higher than the ZM-H, Car-H, ZM + Pio, Car + Pio and Dexa groups (p<0.05 to p<0.001). In the ZM-L group, lymphocyte count was also lower than the PQ group (p<0.05). However, eosinophil counts only in the ZM-H and Car-H groups were lower than the PQ group (both, p<0.05) (Figures 2-4). The total number of WBCs amd the numbers of neutrophils, and lymphocytes in the groups treated with high dose of the extract and Car were lower than their low doses (p<0.05 to p<0.001). However, no significant difference was seen between the two doses of the extract and Car in the number of monocytes and eosinophils, (Figures 2 and 3). 

Only the number of neutrophils and lymphocytes in the Pio group, ZM-L and Car-L was higher compared to the Dexa group (p<0.01 to p<0.001), (Figures 2 and 3). The total number of WBCs in the ZM + Pio and Car + Pio group was less than the Pio, low doses Car and the ZM (p<0.05 and p<0.01). The number of neutrophils and lymphocytes in the treatment groups of ZM + Pio and Pio + Car was less than Pio, ZM-L, and Car-L groups (p<0.05 and p<0.01 for neutrophils, p<0.01 and p<0.001 for lymphocytes) (Figures 2-4).

### Markers of oxidative stress

**Figure 5:**
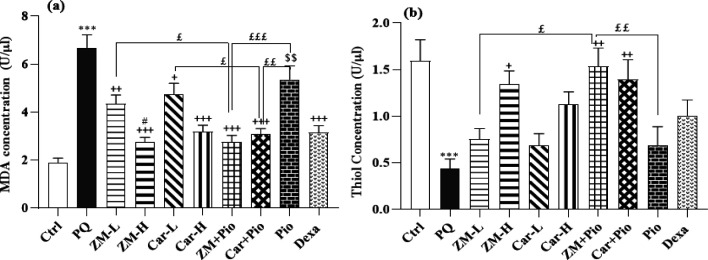
The concentration of MDA (a) and Thiol (b) in the serum of control (Ctrl) group, the group exposed to paraquat (PQ, 54 mg/m^3^), groups treated with two doses of thyme extract (200 and 800 mg/kg/day, ZM-L and ZM-H), two doses of carvacrol (20 and 80 mg/kg/day, Car-L and Car-H), pioglitazone (5 mg/kg/day, Pio) as well as a low dose of extract + pioglitazone (ZM + Pio) and low dose carvacrol + pioglitazone (Car + Pio), dexamethasone (0.03 mg/kg/day, Dexa). (Number of animals for each group = 6). The results are shown as Mean ± SEM. The results of the groups were compared using ANOVA and Tukey statistical tests. Statistical differences between PQ *vs* control group: *** p<0.001, Statistical differences between PQ *vs* treatments groups: + p<0.05, ++ p<0.01 and +++ p<0.001, Statistical differences between Dexa *vs* treatment groups: $$ p<0.01, Statistical differences between combination groups *vs* ZM-L, Car-L and Pio groups: £ P<0.05, ££ P<0.01 and £££ P<0.001. Statistical differences between low *vs* high dose groups of extract and carvacrol: # p<0.05.

**Figure 6:**
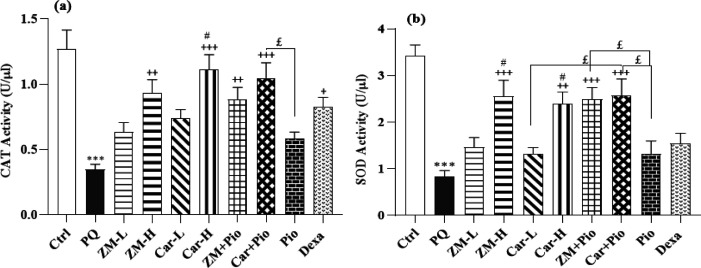
The activity of CAT (a) and SOD (b) in the serum of paraquat (PQ, 54 mg/m^3^), control (Ctrl) and groups treated with two doses of thyme extract (200 and 800 mg/kg/day, ZM-L and ZM-H), two doses of carvacrol (20 and 80 mg/kg/day, Car-L and Car-H), pioglitazone (5 mg/kg/day, Pio) as well as a low dose of extract + pioglitazone (ZM + Pio) and low dose carvacrol + pioglitazone (Car + Pio), dexamethasone (0.03 mg/kg/day, Dexa). (Number of animals for each group = 6). The results are shown as Mean ± SEM. The results of the groups were compared using ANOVA and Tukey statistical tests. Statistical differences between PQ *vs* control group: *** p<0.001, Statistical differences between PQ *vs* treatments groups: + p<0.05, ++ p<0.01 and +++ p<0.001. Statistical differences between combination groups *vs* ZM-L, Car-L and Pio groups: £ P<0.05, Statistical differences between low and high dose groups of extract and Carvacrol: # p<0.05.

An increase in the serum level of MDA but decreased total thiol groups, and activities of SOD and CAT enzymes were observed in the PQ group compared to the control group (all cases, p<0.001), (Figures 5 and 6). MDA concentration in all treated groups except Pio was lower than the PQ group (p<0.05 to p<0.001). Increased total thiol in the ZM + Pio, Car + Pio, and ZM-H and increased CAT and SOD activities in the ZM-H, Car-H, ZM + Pio, Car + Pio, ZM-L, and Dexa groups were observed, except SOD in the Pio, Dexa groups (p>0.01 to p>0.001) (Figures 5 and 6). The amount of MDA was lower in the ZM-H group compared to the ZM-L group (p<0.05). The activities of SOD and CAT enzymes were higher in the ZM-H group compared to the ZM-L group (p<0.05) (Figure 5 and 6). The amount of MDA in the Dexa group was lower than the Pio group (p<0.01), (Figures 5 and 6). The amount of MDA in the serum of the ZM + Pio and Car + Pio groups was lower than the Pio, ZM-L, and Car-L groups (p>0.05 for ZM-L and Car-L, p<0.01 to p<0.001 for Pio). Total thiol, CAT, and SOD levels were higher in combined treatment groups compared to the Pio, ZM-L, and Car-L groups (p<0.05 to p<0.01) (Figures 5 and 6).

### The levels of IL-10 and TNF-α

**Figure 7:**
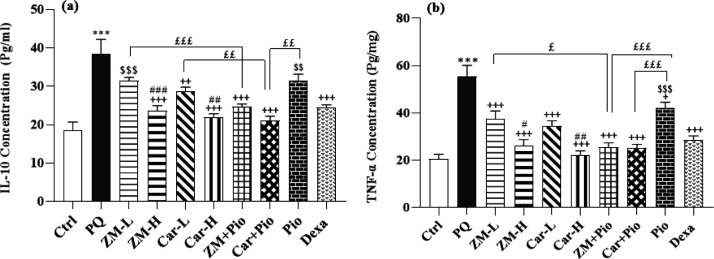
Cytokine concentration of IL-10 (a) and TNF-α (b) in the serum of control (Ctrl) group, the group exposed to paraquat (PQ, 54 mg/m^3^), groups treated with two doses of thyme extract (200 and 800 mg/kg/day, ZM-L and ZM-H), two doses of carvacrol (20 and 80 mg/kg/day, Car-L and Car-H), pioglitazone (5 mg/kg/day, Pio) as well as a low dose of extract + pioglitazone (ZM + Pio) and low dose carvacrol + pioglitazone (Car + Pio), dexamethasone (0.03 mg/kg/day, Dexa). (Number of animals for each group = 6). The results are shown as Mean ± SEM. Statistical differences *vs.* control group: *** p<0.001. Statistical differences *vs* PQ group: + p<0.05 and +++ p<0.001. Statistical differences *vs* Dexa group: $$ p<0.01 and $$$ p<0.001. Statistical differences between combination groups *vs* ZM-L, Car-L and Pio groups: £ p<0.05, ££ p<0.01 and £££ p<0.001. Statistical differences between low *vs* high dose groups of extract and carvacrol: # p<0.05, ## p<0.01 and ### p<0.001.

 The levels of TNF-α and IL-10 in the control group were lower than the PQ group (p<0.001 for both cases) (Figure 7). The levels of IL-10 and TNF-α in all treated groups were lower than the PQ group except IL-10 level in the ZM-L and Pio groups (p<0.05 to p<0.001) (Figure 7). The levels of IL-10 and TNF-α in the ZM-H and Car-H were lower than the ZM-L and Car-L group, respectively (p<0.01 to p<0.001 for IL-10 and p<0.05 to p<0.01 for TNF-α), (Figure 7). The level of IL-10 in the Pio and ZM-L and that of TNF-α in the Pio group were higher than the Dexa group (p<0.01 to p<0.001 for IL-10 and p<0.001 for TNF-α) (Figure 7). The level of IL-10 and TNF-α in the group treated with ZM + Pio were lower than the ZM-L and Pio groups except IL-10 in the Pio group (p<0.05 to p<0.001). On the other hand, in the group treated with Car + Pio were lower than the Car-L and Pio groups except TNF-α in the Car-L group (p<0.01 to p<0.001).

## Discussion

Inhalation of PQ in rats resulted in an amplification of both differential and total WBC counts, as well as elevated levels of MDA, TNF-α, and IL-10 in the serum. Conversely, the levels of total thiol group and the activities of CAT and SOD were diminished. Consequently, the inhalation of PQ led to the manifestation of systemic oxidative stress and inflammation. A recent study showed that i.p. administration of PQ (30 mg/kg) in mice caused oxidative stress associated with enhanced MDA level and reduced glutathione and CAT in the lung tissue (Mirzaee et al., 2019). Also, systemic oxidative stress and inflammation induction by PQ through raising intracellular cyclic GMP level and activation of guanylate cyclase was reported (Giri and Krishna, 1979). The molecular and cellular mechanisms of PQ toxicity have not yet been completely explained, but animal studies have shown that one of the most important mechanisms of PQ poisonous is oxidative stress. In the body, PQ is converted into a cationic radical, which due to the production of free radicals through the NADPH-dependent one-electron reduction process, ultimately leads to tissue damage (Keeling and Smith, 1982). Through the formation of reactive species (ROS), PQ leads to an increase in free radicals and a decrease in the reserves of reduced GSH and an increase in oxidized glutathione (GSSG), which causes cell death and fibrosis (Zhao et al., 2016). The outcomes of previous studies confirm the findings of this research with PQ inhalation, which is more consistent with farmers' exposure to this herbicide. 

Treatment with Car, ZM, Dexa, and Pio similarly decreased differential and total WBC counts, MDA, IL-10 and TNF-α, but raised thiol content, CAT, and SOD activities in the serum of mice exposed to PQ. Therefore, the findings of the present research indicated that the applications of ZM, Car, Pio, and Dexa improved oxidative stress and systemic inflammation caused by PQ (Koeberle and Werz, 2018). The effects of ZM-L and Car-L were lower than ZM-H and Car-H offering a dose-dependent effect of the extract and Car. Regarding the effect of thyme extract and Car on antioxidant factors, a previous study support the findings of the present study (Khazdair et al., 2018). Ahmadipour et al. (2015) showed that Shirazi thyme extract reduced liver damage and enhanced the activity of antioxidant enzymes in a dose-dependent manner (Ahmadipour et al., 2015) Car is one of the effective ingredients in thyme extract, which can reduce oxidative stress in many diseases. For example, Car reduced some inflammatory mediators and MDA in the animal model of COPD (Mahtaj et al., 2015). 

The results indicated a low effect of a low dose of Pio on systemic oxidative stress and inflammation induced by PQ inhalation. The effects of PQ-induced systemic oxidative stress and inflammation were demonstrated in a previous study on Pio (Amin et al., 2021a). The inhibition of myeloperoxidase function, as well as the suppression of TNF-α protein and mRNA manifestation, were effectively accomplished by the administration of Pio. Within the renal, neural, and hepatic tissues, the activation of PPAR-γ receptors has been observed to mitigate the detrimental effects of oxidative stress and inflammatory responses (Al Rouq and El Eter, 2014; El-Sheikh and Rifaai, 2014; Zou et al., 2013). 

In the combined Pio + ZM-L and Pio + Car-L groups improvement effect in most studied measured variables was higher than Pio, ZM-L, and Car-L alone. These findings can indicate that ZM extract and Car may improve systemic inflammation and oxidative damage caused by exposure to PQ through activation of PPAR-γ receptors. Previous studies have shown that Car can act as an activator of the PPAR-γ receptor (Hotta et al., 2010) which supports this effect. In a previous study, similar findings were shown, when the treatment effects of ZM and Car were evaluated on oxidative stress and systemic inflammation by treating animals for 16 days after the end of PQ inhalation which also supports this suggestion (Amin et al., 2021a). Despite this, further research should be conducted to assess how ZM and Car affect systemic injury triggered by PQ inhalation with a PPAR-γ receptor antagonist to corroborate the mechanism of action of the plant and its constituent.

Dexa as a well-known anti-inflammatory drug also indicated similar effects to ZM, Car, and their combination on systemic changes induced by PQ inhalation which may support the anti-inflammatory mechanism of the effects of ZM and Car. Thus, the findings of the current and previous studies suggested therapeutic effects of ZM, Car, Pio, and Dexa on systemic oxidative stress and inflammation caused by PQ (Koeberle and Werz, 2018).

One of the constraints of the present investigation is the lack of thorough examination regarding the impact of ZM or Car on the PPAR-γ receptor. This could be accomplished by evaluating the consequences of ZM and Car in the presence or absence of PPAR-γ receptor antagonist medications, a task that should be undertaken in forthcoming studies. Furthermore, it is essential to explore the effects of ZM or Car on apoptosis and the expression of cytokine genes. Examining different doses of ZM and Car is also of scientific values.

The finding of this study indicates the preventive effect of ZM and Car on systemic oxidative stress and inflammation caused by PQ inhalation in rats. The combination of ZM, or Car with Pio showed a positive impact on PQ inhalation-induced systemic changes. The synergistic effect between Pio with ZM or Car suggests the involvement of the PPAR-γ receptor in the effects of thyme and its constituent but more studies are required to support this suggestion.
